# Single-molecule fluorescence microscopy review: shedding new light on old problems

**DOI:** 10.1042/BSR20170031

**Published:** 2017-07-21

**Authors:** Sviatlana Shashkova, Mark C. Leake

**Affiliations:** 1 Department of Physics, Biological Physical Sciences Institute (BPSI), University of York, York YO10 5DD, U.K.; 2 Department of Biology, Biological Physical Sciences Institute (BPSI), University of York, York YO10 5DD, U.K.

**Keywords:** fluorescence, microscopy, single-molecule, super-resolution

## Abstract

Fluorescence microscopy is an invaluable tool in the biosciences, a genuine workhorse technique offering exceptional contrast in conjunction with high specificity of labelling with relatively minimal perturbation to biological samples compared with many competing biophysical techniques. Improvements in detector and dye technologies coupled to advances in image analysis methods have fuelled recent development towards single-molecule fluorescence microscopy, which can utilize light microscopy tools to enable the faithful detection and analysis of single fluorescent molecules used as reporter tags in biological samples. For example, the discovery of GFP, initiating the so-called ‘green revolution’, has pushed experimental tools in the biosciences to a completely new level of functional imaging of living samples, culminating in single fluorescent protein molecule detection. Today, fluorescence microscopy is an indispensable tool in single-molecule investigations, providing a high signal-to-noise ratio for visualization while still retaining the key features in the physiological context of native biological systems. In this review, we discuss some of the recent discoveries in the life sciences which have been enabled using single-molecule fluorescence microscopy, paying particular attention to the so-called ‘super-resolution’ fluorescence microscopy techniques in live cells, which are at the cutting-edge of these methods. In particular, how these tools can reveal new insights into long-standing puzzles in biology: old problems, which have been impossible to tackle using other more traditional tools until the emergence of new single-molecule fluorescence microscopy techniques.

## Introduction

### Why do we care about detecting single molecules in cells?

Experimental investigations in the life sciences have traditionally been performed on a population ‘ensemble average’ level. An example of this is the use of cell cultures, which contain a population of many thousands of cells. A cell population is, in general, intrinsically heterogeneous, even if cells are genetically identical. In other words, different cells exhibit a range of different physical, chemical and biological properties. Such heterogeneity is potentially valuable at a level of the originator species, in that they allow rapid adaptation in a dynamic, fluctuating environment, and so may impart a biological advantage to the ultimate survival of the species [1–3]. Using a population signature as a metric for the physical or chemical status of different cellular parameters is valuable at one level, since it averages out the observations of potential minor and anomalous cells in that population, in effect smoothing out the ‘noise’. However, the main problem with this approach is that there may be valuable information hidden in this ‘noise’; we run the risk of losing potentially useful data concerning biologically relevant heterogeneity. We potentially limit the extent to which we can investigate ‘subpopulations’ [4,5].

For example, ensemble average analysis will not pinpoint the drug-resistant bacteria or cancer cells in a general cellular population. When subpopulations are identified, the only way to determine which cells contribute to which group, hence, to separate competing signals, is to analyse the whole population cell-by-cell [6,7]. Population heterogeneity can arise due to environmental alterations affecting the soft matter of biological material [8], as well as through genetic variation that affects gene expression and can invoke fluctuations in various cellular components [9]. Differences in transcriptional regulation affect signal transduction pathways and hence responses to various stress factors, such as pH and oxidative stress. Therefore, an ideal single-cell experiment should be performed under precise environmental control. Moreover, the age of a cell and its phase in the cell cycle may also significantly influence the cell response [4].

Even an apparently simple unicellular organism represents a heterogeneous system on a molecular level [10]. Analysis of the ensemble average of molecular properties results in loss of information concerning any molecular heterogeneity, and may ultimately lead to misinterpretations of the underlying physiological relevance of subpopulations of molecules [11]. Focusing on molecules as the minimal ‘functional’ units in a biological system, single-molecule biophysics research has an important impact on a range of fields of biological investigation. These include fields where biological complexity is rife, such as medical immunology, synthetic and systems biology, but also several others at a more basic mechanistic level, ultimately through an ability to enhance both the effective spatial and temporal resolution of data [11]. Modern techniques [12] enable, for example the probing of the cellular signal transduction dynamics directly [13], which facilitates a deeper and more precise understanding of important biological processes, e.g. the human immune response, gene expression and cellular differentiation. One of the most important techniques used currently in single-molecule biophysics research is, unquestionably, fluorescence microscopy [14,15].

Identification and investigation of molecular subpopulations within the cell enables us to study not only cellular responses but also the precise underlying molecular mechanisms. Arguably, the first clear demonstration that single-molecule fluorescence microscopy could yield insight which were genuinely unanticipated from bulk ensemble average measurements was reported in 1998. Here, the researchers used the native photoblinking behaviour of the common metabolite FAD inside a binding site of the enzyme cholersterol oxidase to demonstrate that its activity could be affected by a type of ‘molecular memory’ stored in the molecular confirmation [16]. Single-molecule fluorescence microscopy approaches since then have uncovered many fundamental molecular scale biological processes that were previously not studied primarily due to the limitations imposed by population methods, including studies of the bacterial flagellar motor rotation [17–21], protein folding, translocation and movement [11,22–25], signal transduction [26], biopolymer mechanics [27–32], DNA replication and remodelling [33–37], oxidative phosphorylation [38–41], as well as biomedically relevant areas such as the probing of processes relating to infection and general pathology [42–44], cell division mechanisms [45], mitochondrial protein dynamics [46], viral infection processes [47], endocytocis and exocytosis pathways [48], osmolarity receptor dynamics [49], cell wall synthesis [50], and structural dynamics of DNA [51]. This list above should not be taken as exclusive nor exhaustive, but rather we present it here to exemplify the very wide range of biological processes to which single-molecule fluorescence microscopy tools have been applied.

One of the primary requirements for all the single-cell/single-molecule approaches is the ability to faithfully detect small signals over sometimes relatively large noise levels [52]. Combining improvements in a range of different approaches, such as minimizing the sample volume, engineering better photostability for newer variants of fluorescent proteins, and improving the sensitivity of camera detectors, have resulted in higher detection levels of photon signals for fluorescence emission, though still there are limitations due to poor signal-to-noise ratios when sampling at very high imaging rates. Various analytical tools have been developed to improve the signal-to-noise ratio, such as automated methods of ‘segmentation’ of cellular images [53,54], robust software algorithms for the tracking of fluorescently labelled molecules [55–57], and stoichiometry analysis of molecular complexes which those tracked molecules form. We steer the reader to recent comprehensive reviews that discuss these different approaches on how to increase the fidelity of signal detection over background noise [52,58].

### Fluorescence and fluorescent proteins

**Figure 1:**
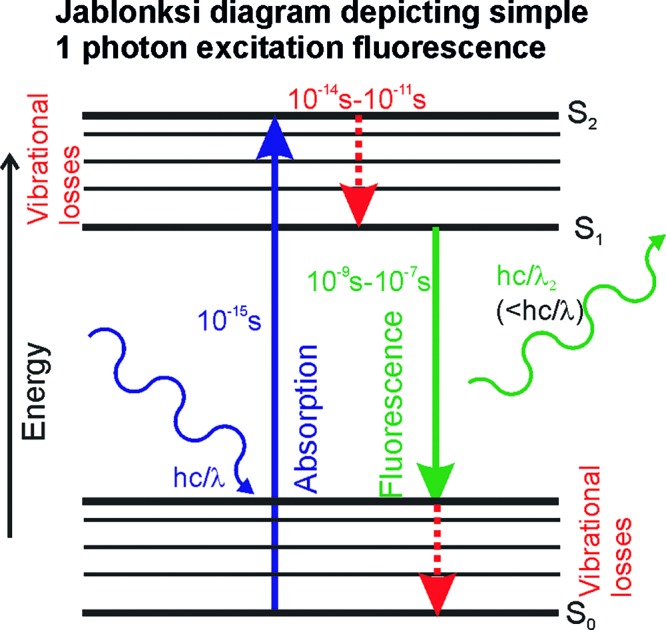
Jablonski diagram An electron of a fluorophore at the ground state (S_0_) receives energy from the absorption of a single photon of light which results in an excitation transition to a higher energy state (absorption). When the excited electron relaxes to the ground state, following vibrational losses, energy, lower than the incident photon and thus with a higher wavelength, is emitted as a single photon which causes fluorescence.

The physical process of fluorescence occurs when a photon of light is absorbed by a ‘fluorophore’, which may be an atom or a molecule, and consequently re-emitted as a photon with a longer wavelength. The loss of energy occurs due to vibrational processes which result from oscillations between the atomic/molecular orbitals due to the perturbation of a different negative electron charge distribution relative to the positively charged nucleus. Upon standard ‘single photon excitation’, light absorption of a single photon (of light) occurs which results in a ground state electron in the fluorophore undergoing an excitation transition to a higher energy state, in a process characterized by a time scale of ∼10^−15^ s. Following this relatively transient state, the excited electron loses energy through vibrational losses over a time scale of 10^−14^–10^−11^ s. The electron then undergoes an energy transition back to the ground state, characterized by a time scale of 10^−9^–10^−7^ s, accompanied by photon emission, whose wavelength is longer than the incident wavelength (i.e. has a smaller associated energy). Jablonski [59] described the different energy states and transitions between them in a useful pictorial form called Jablonski diagram (Figure 1). Although the physical process of fluorescence was properly formulated by the British scientist Stokes et al. [60], it was more than half a century later that the first operational fluorescence microscope was developed, reported in 1911, which obtained the relatively standard design as we know it today only in 1967 [61].

In 2008, the Nobel Prize in Chemistry was awarded jointly to Osamu Shimomura, Martin Chalfie and Roger Y. Tsien for the ‘discovery and development of green fluorescent protein, GFP’ [62]. GFP had been isolated from the jellyfish *Aequorea victoria*, described in an article in 1962 [63]. A step change came when the *GFP* gene was sequenced in the early 1990s, accompanied by developments in molecular cloning technologies enabling the integration of its DNA directly into DNA in other organisms. Nowadays, it is an invaluable tool which is widely used as a fluorescent tag and can be relatively easily integrated into the genome. GFP is a β-barrel protein consisting of 11 β-sheets and an α-helix, composed of 238 amino acids residues in total. The wild-type GFP chromophore is encoded by the Ser^65^-Tyr^66^-Gly^67^ sequence which forms a heterocyclic photoactive state spontaneously through the processes of intramolecular autocatalytic rearrangement and subsequent oxidation [64]. This final oxidation stage is crucial for the protein to function as an active fluorophore.

Numerous mutations of wild-type GFP have now been generated, with one of the principle aims of improving its biophysical characteristics. Photostability and fluorescence output increases were achieved by using an S65T mutation [65], while the A206K was developed to prevent self-oligomerization [66], and various colour mutations were added, including, for example blue Y66H, cyan Y66W and yellow T203Y [67] variants. Standard fluorescent proteins will undergo irreversible photobleaching after a characteristic time interval when excited to fluorescence, most likely to be due to the accumulation of free radicals in the surrounding water solvent formed from the lysis of water molecules upon absorption of photons of light, and their subsequent chemical damage to the fluorescent protein structure. Standard fluorescent proteins cannot therefore be tracked longer than their photobleaching point, which thus limits their application in long time scale experiments.

Certain newly engineered fluorescent proteins, e.g. mEos [68], Dendra [69] and KikGR [70] can be photoactivated and undergo irreversible photoconversion from green to red emitting state upon irradiation with UV light [71,72]. Although such approaches potentially can appear to extend the lifetime of a tracking experiment in which proteins can be photoconverted before they bleach, there is no intrinsic improvement as such to photostability in these proteins. Monomeric forms of these proteins [73,74] as well as different variants of photoconvertible proteins with enhanced features have also been designed. For example, mMaple protein exhibits reversible photoconversion under certain conditions [75], with yellow-to-cyan (EYFP-to-CFP) photoconversion upon green light illumination [76], and cyan-to-green photoswitch of PS-CFP2 [18]. The fluorescent protein mOrange undergoes orange-to-red activation upon illumination with blue light (typically using the common laser line with wavelength 488 nm), which is thus less harmful for live cells compared with UV-convertible proteins in regard to photodamage effects [77]. Photoconversion can be used stroboscopically to divide up the finite photon budget prior to photobleaching (i.e. acquiring fluorescence images over extended time intervals instead of continuously illuminating samples), which has been used to monitor complex live samples such as developing embryos for up to several hours [78]. Another type of fluorescent protein, phytochrome-based near IR fluorescent proteins (iRFP), has been developed recently [79]. Compared with conventional fluorescent proteins, such as GFP, iRFP has a higher effective signal-to-noise ratio and allows imaging deeper into tissues due to smaller elastic scattering effects at higher wavelengths of electromagnetic radiation, relevant for applications in live-animal or excised-tissue models.

Some fluorescent proteins have a characteristic time over which they change their emission wavelength from blue to red based on the chromophore maturation time. Such proteins can be used, therefore, as fluorescent timers, such as to study protein transport. For example, an mCherry-derived monomeric variant with various timing behaviours has been used for probing the kinetics of protein trafficking [80].

Fluorescent probes may be added to a protein of interest directly or via linkers, such as SNAP- and HALO-tags. Here, the encoding DNA for a protein probe is first genomically fused next to the protein under investigation, technologically similar to the approach used in developing fluorescent protein fusion constructs. In most applications of HALO/SNAP, this probe consists of a DNA repair protein (for SNAP) or a haloalkane dehalogenase enzyme (for HALO) [81,82]. The cell can then be incubated with a secondary probe which is fluorescently labelled with a bright organic dye fluorophore. The secondary probe is designed to bind to the primary protein probe. The use of these tags avoids ‘direct’ fluorescent protein labelling, which might impair their physiological behaviour due to steric hindrance. This methodology enables a far brighter and more photostable fluorophore to be used compared with conventional fluorescent proteins. Since the localization precision improves with the brightness of the fluorophore used (roughly with a reciprocal dependence on the square root of the brightness) the use of a brigher dye facilitates improvements in localization precision for determining the position of individual fluorophores. This method also implies a potential improvement to temporal resolution in ultimately enabling faster sampling for a given spatial localization precision. That being said, the primary probes for SNAP and HALO are themselves reasonably large whose molecular weight is only ∼40% less than that of fluorescent proteins of ∼28 kDa [5], and so a potential steric hindrance effect is still present. Also, the efficiency of labelling during the secondary probe incubation step is sometimes difficult to achieve as the primary protein probe is often not easily accessible, e.g. the primary probe protein is deep inside a cell and thus there are technical issues in how to deliver the secondary probe to these regions. However, this approach has resulted in significant advances in super-resolution imaging of accessible cell surface structures, such as the cell wall architecture of bacteria [83].

## Main techniques and applications of single-molecule fluorescence microscopy

### A potted history of the development of single-molecule fluorescence microscopy

The first report of the inference of the presence of single molecules using fluorescence microscopy came as early as 1961 from the work of Boris Rotman. In that study, the product of an enzyme-catalysed reaction was labelled with a fluorescent dye, and since each single enzyme molecule resulted in the manufacture of several thousand product molecules, this intrinsic ‘chemical amplification’ resulted in the ability to detect a fluorescent signal from aqueous droplets of the reaction solution immobilized on to a mica surface [84] using relatively insensitive camera technology compared with those we use today. The direct detection of single molecules using fluorescence microscopy was first reported in 1976 from the work of Thomas Hirshfeld in which molecules of the protein globulin were labelled with, on an average, several tens of a bright organic dye fluorophore molecule, and these fluorescently labelled particles could be detected as they were flowed past a photodetector in aqueous solution. The first report of detecting single fluorescent dye tags directly, which used a similar experimental approach, came in 1990 [85].

Following these seminal developments, the 1990s brought forth many important developments in regard to improving the spatial resolution of detection of single molecules using fluorescence microscopy. It may be useful for us now to consider some of the basics about optical resolution theory. Fluorophores which are visualized in the ‘far-field’ regime (i.e. there is a distance of several wavelengths of light between the fluorescence source and the detector) exhibit diffraction. In a light microscope, the apertures through which light propagates are in general circular and for these the diffraction pattern which results is known as an Airy ring or disk, the shape of which is determined by the so-called point spread function (PSF) of the microscope system.

The intensity profile of an Airy ring pattern can be described analytically by using a mathematical function called Bessel function – this 2D relation consists of a central circular region of bright intensity surrounded by alternating minima and maxima in concentric rings of increasing radius from the centre. The diffraction angle of the first dark ring, *θ*, mathematically satisfies the equation sin*θ*≈1.22λ_m_/2*r* =0.61λ_m_/*r*, where *r* is the radius of the circular aperture in question and λ_m_ is the wavelength of the light in the imaging medium. If the circular component is an objective lens of focal length *f*, then the lateral distance *d* in the image plane from the optic axis to the first dark ring is given by the relation *f*sin*θ*_max_, where *θ*_max_ is the maximum allowed diffraction angle corresponding to the first dark ring emerging from the circular aperture. If the imaging medium has a refractive index *n*, then λ_m_ = λ/*n* where λ is the wavelength of the light in vacuum. Rearranging these relations thus indicates that *d* =0.61 λ/nsin*θ*_max_ =0.61 λ/NA, where NA is the numerical aperture of the objective lens.

If there are two Airy ring patterns overlapping such that the centre of one just overlaps with the first dark ring of the other, then their separation will be equal to *d*. This is the basis of the ‘Rayleigh criterion’ of the optical resolution limit. It approximates to the minimal distance at which two points can be *distinctly* detected in the far-field regime in a light microscope. In 1873, Abbe [86] described that the optical resolution of the light microscope is limited by the diffraction properties of light, through the formulation (known as ‘Abbe’s limit’) of λ/2NA, by consideration of *rectangularly* shaped apertures in a diffraction grating (as opposed to a circular aperture), and for these the equivalent diffraction angle is given as λ_m_/2*D* where *D* is the aperture width.

Optical imaging techniques which render spatial information at a precision better than the optical resolution limit are known as ‘super-resolution’ microscopy methods. These have added an exceptional level of insight into challenging biological questions, exemplified in 2014 when Eric Betzig, Stefan W. Hell and William E. Moerner were jointly awarded the Nobel Prize in chemistry ‘for the development of super-resolved fluorescence microscopy’ [87]. Development of a range of super-resolution techniques have been invaluable for single-molecule fluorescence microscopy, enabling scientists to break the optical resolution limit to study the functional localization and interactions of biological substructures at the level of single molecules down to nanoscale precision [10]. Today, these varied super-resolution methods enable us to obtain genuinely new insights into fundamental biological mechanisms which have been long-standing questions in the field: the ‘old problems’ of the title of this review that were previously intractable due, primarily, to technical limitations imposed by conventional ensemble average methods.

Arguably, the simplest way to achieve super-resolution is to avoid imaging in the far-field regime, *per se*, but rather perform *near-field* imaging (i.e. where the fluorescent source and detector are separated by less than a few wavelengths of light) since, then one is not subjected to significant optical diffraction effects. In this regard, the first experimental application of super-resolution light microscopy was reported in 1981, as the technique called total internal reflection fluorescence (TIRF) microscopy [88]. Although resulting fluorescent samples being subjected to standard diffraction-limited resolution in the lateral plane of the microscope (i.e. the focal plane), TIRF uses near-field excitation axially by generating an evanescent field which delimits this axial illumination to a length which is shorter than the standard optical resolution limit. The first single-molecule biological application of this technology was reported in 1995 involving *in vitro* experiments to monitor ATP turnover by single myosin molecules [89], while the first single-molecule fluorescence microscopy imaging in live cells also used TIRF, reported in 2000, which investigated epidermal growth factor (EGF) receptors and ligand binding using fluorescently labelled EGF [90].

Another early developed near-field, single-molecule fluorescence technique used scanning near-field optical microscopy (SNOM or NSOM). Here, the laser excitation field operates over a length scale shorter than the optical resolution limit in being limited by the size of the probe tip down to just approximately 10 nm, first demonstrated on single fluorescent molecules in 1993 [91]. A modification of this approach enabled excitation of a donor fluorophore in the non-radiative technique of single-molecule FRET (smFRET), reported first in 1996 [92]. Here, not only is the excitation field smaller than the optical resolution limit, but also FRET only operates over a length scale comparable with the physical size of the molecular orbitals of the donor and acceptor fluorophore dyes employed of approximately 0–10 nm.

Another commonly used super-resolution method today involves localization microscopy. This is performed in the far-field regime and relies on the fact that the centre of an Airy disk pattern is the best estimate for where the actual fluorescence-emitting dye molecule is in space. Thus, if Airy disk patterns from a population of many dye molecules are separated by greater than the optical resolution limit then, we can apply mathematical fitting methods to estimate where their fluorescence emission ‘centres’ actually are. This approach was used to monitor the diffusion of single fluorescent molecules to a precision roughly to an order of magnitude better than the optical resolution limit, first reported in 1996 by Schmidt et al. [93]. Here, a fitting algorithm was used which approximated the central intensity of the Airy disk pattern using a Gaussian function in a method applied to micron-sized beads used as a probe on single kinesin molecule translocating on microtubules tracks, reported from the lab of Sheetz et al. in 1988 [94].

**Figure 2:**
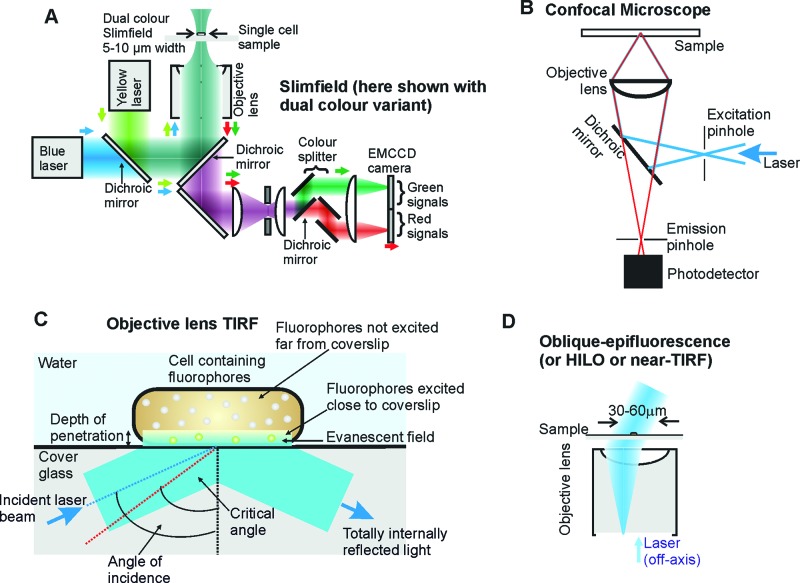
Schematic representation Of: (**A**) **Slimfield imaging**; (**B**) a **confocal microscope**; (**C**), **TIRF** showing the illumination of fluorophores close to the glass coverslip surface (detailed explanation is provided in the text); (**D**) **HILO microscopy**.

There have been myriad super-resolution studies published to date which utilize localization microscopy approaches, but arguably the most recent developments with this basic method have involved improvements using probabilistic methods of single particle tracking. For example, the so-called Bayesian approaches to infer the detection and tracking of fluorescently labelled particles [95], and improvements to the speed of tracking. For instance, techniques which reduce the size of the illumination area of excitation, like slim-field or narrow-field fluorescence microscopy, have been developed [96]. Such methods can enable tracking of single fluorescent proteins over very rapid millisecond time scales which significantly reduces motion blur of diffusing fluorescently labelled molecules in different compartments of live cells (Figure 2A), especially in the cytoplasm in which the viscosity is relatively low and so the rate of diffusion is relatively high [33]. These rapid imaging single-molecule fluorescence microscopy techniques may also be combined with convolution analysis of live cell fluorescence images to determine the copy number of proteins in a single cell, and indeed in separate cellular compartments [97]. Most super-resolution techniques, however, are mainly based on conventional fluorescence and confocal microscopy principles [98], but have resulted in huge advances in our knowledge of the biosciences, in particular concerning how processes operate in functional, living cells.

This ‘intrinsic’ optical resolution limit can also be broken, however, even in a far-field regime. In the 1980s, a Russian scientist Okhonin [99] patented the first super-resolution microscope based on ‘stimulated emission depletion (STED)’, however, experimentally the principle was first demonstrated only later by Hell et al. [100], apparently unaware of Okhonin’s earlier insight judging from the lack of reference to Okhonin’s patent.

In the sections below, we outline some of the specific details concerning the modern light microscopy techniques which enable single-molecule fluorescence-based detection in particular.

### Confocal microscopy

The conception of confocal microscopy (Figure 2B) is often attributed to Minsky [101,102], who published an initial patent for a confocal microscope in 1961. This method, also called confocal laser scanning microscopy (CLSM), enables in-effect optical sectioning through a biological sample. The word ‘confocal’, implying ‘having the same focus’, refers to the presence of two pinholes which are conjugated in the same image plane. One of them is used for spatial filtering of the excitation laser beam by removing side lobes at a position where the laser beam is focused, and the other for the emitted light path, which eliminates a significant proportion of stray signals coming from above or below the focal plane. The first practical working confocal microscope was built by the pioneering efforts of Eggar and Petran [103], whose first biological application was reported in 1967 to visualize unstained nerve cells in the brain.

The basic method for confocal microscopy involves scanning of the specimen by moving either the stage in vertical and horizontal directions (Minsky’s method) or the laser beam in more modern systems [104]. Confocal microscopy can significantly improve the effective signal-to-noise ratio for detection by removing out-of-focus fluorescence. For instance, confocal fluorescence microscopy was used in studies of DNA repair processes through observing bubble DNA/GFP-tagged nucleotide excision repair (NER) protein interactions under physiological conditions [105]. Development of a novel video-rate confocal microscope allowed monitoring, the diffusion of *Dictyostelium discoideum* cAMP receptors on basal and apical surfaces [106]. Many recent single-molecule studies utilize a confocal microscope primarily simply to generate a ‘confocal excitation volume’ (i.e. a diffraction-limited, 3D-focused laser ‘spot’ in the sample) to act as the illumination source to excite fluorescently tagged molecules which are then detected by other imaging techniques. Several attempts to improve resolution and signal-to-noise ratio of CLSM have been made recently, including the so-called Airy scan in which each pixel detector element on a camera can be treated as a pinhole, which has enabled, for example, the determination of the shape and dimensions of virus particles [107,108].

### TIRF

TIRF is now one of the most frequently used imaging methods in single-molecule fluorescence microscopy. The method is based on total internal reflection of incident excitation light from a glass–water interface, such as between a glass coverslip/slide and a water-based physiological buffer (Figure 2C). When light which is incident on an interface in refractive index, *n*, such as that between water (*n*=1.33) and glass (*n*=1.52), then refraction of the beam will in general occur at angles of incidence not normal to the interface, deviating the direction of the light, unless the angle of incidence is high enough such that the refraction angle is equivalent to 90° or more. The angle of incidence at which the angle of refraction is exactly 90° is known as the ‘critical angle’, and is simply given by the relation: sin^−1^(*n_water_*/*n_glass_*) or approximately 62°. At angles of incidence greater than this, total internal reflection of the incident beam occurs at the interface, instead of transmission through the water. A caveat of this is that some of the light is allowed to extend beyond the interface into the water as an ‘evanescent field’, whose intensity falls off exponentially with distance from the glass/water interface.

Although the evanescent field is a continuum in space into the water side of the interface, the angle of incidence in TIRF microscopes is often set to allow much of the intensity in the evanescent field to be limited to the first approximately 100 nm beyond the coverslip/slide surface. This is defined as the depth of penetration of the field or the axial distance over which the intensity drops off by a factor of *e*. In reality, this depth of penetration can actually be adjusted over a wide range. ‘Objective lens TIRF’, which is the most commonly employed mode of TIRF operation currently, uses a single objective lens to steer the incident beam and collect fluorescence emissions. Here, the depth of penetration can be set to be as small as approximately 30 nm for very high NA objective lens (e.g. NA =1.65) or can be made arbitrarily larger (extending in principle to infinity).

TIRF in effect delimits the excitation field to result in selective illumination and excitation of fluorophores that are positioned close to the coverslip/slide surface (in practice, this ‘close distance’ can be approximated as being, very roughly, the depth of penetration itself). Thus, TIRF is particularly valuable for identifying single fluorescently labelled molecules integrated into cell membranes, for cells immobilized on to a glass slide/coverslip. Due to the fact that TIRF detects only minimal signals from the out-of-focus regions, the signal-to-noise ratio is significantly improved, enabling better contrast for detecting single molecules [88]. In life science research, TIRF is often used in studies of kinetic properties on a single-molecule level in cell membranes, which is of particular importance in cellular signalling and vesicle trafficking research. For example, using TIRF microscopy the dynamics of the entire cascade of lipopolysaccharide transfer on to toll-like 4 receptor/myeloid differentiation factor 2 was reconstructed [109]. Moreover, TIRF can also be used in single-molecule electrochemistry. Thus, the group of Bo Zhang discovered that mesoporous silica reduces the rate of diffusion of fluorogenic redox molecules, enabling observation of single redox events. The study was carried out on fluorescent resorufin allowing analysis of adsorption, desorption and redox events by TIRF molecules on transparent ITO electrodes coated with mesoporous silica [110].

TIRF is widely used in cytoskeleton assembly studies. Thus, TIRF provided novel insights into actin filament dynamics and network architecture on a single filament [111] as well as single-molecule detection sensitivity for the visualization and analysis of capping and uncapping of individual actin filaments in vertebrates [112]. Single-molecule TIRF revealed that, depending on the filament age, trafficking of myosin molecules results in sorting to different F-actin networks [113]. Enabled/vasodilator-stimulated phosphoprotein (Ena/VASP) regulates actin network assembly by interacting with actin filaments. TIRF revealed multiple modes of VASP–F-actin interactions underlying mechanisms of VASP action [114]. A detailed application of TIRF and other single-molecule methods on actin assembly and disassembly have been described recently [115].

‘Near TIRF’ (also known as HILO or oblique-angle epifluorescence), enhances imaging contrast but enables greater depth of imaging for non-surface processes (Figure 2D). HILO techniques can therefore be very beneficial in single-molecule studies in live cells. Both HILO and TIRF can be used for visualization of molecular diffusion. For example, these techniques were applied in studies of the behaviour of CheY, a protein used by *Escherichia coli* in its chemotactic response, revealing movements among chemoreceptor clusters, flagellar motors and switch complexes [116]. Dynamic properties of the plasma membrane were described by single-molecule tracking visualized by TIRF. The mobility of some proteins in the plasma membrane was identified by the work of Kusumi et al. as being putatively confined hop diffusion which does not depend upon the extracellular matrix and extracellular domains of proteins [117]. Several years earlier, Kusumi et al. used the same approach to examine MHC class II protein diffusion [118].

TIRF imaging of a human serotonin transporter (SERT) was used to study its diffusion at the plasma membrane and endoplasmic reticulum (ER) revealing stable and highly mobile fractions of SERT at the ER [119]. Later, various oligomeric forms of SERT were identified. In the same study, a combination of TIRF with ‘thinning out clusters while conserving stoichiometry’ (TOCCSL) was used to determine the oligomeric states of the proteins in both the compartments. However, TOCCSL can be used for studies of the mobile protein fractions only [120].

Molecular transport based on kinesin and dynein movements along microtubule filaments is fundamental for various cellular processes such as mitosis, meiosis, proteins, mRNA and organelle cargos, which are crucial for survival and morphogenesis [121]. Significant mechanistic insights into kinesin-based transport, for example, were obtained by single-molecule TIRF imaging of single GFP-labelled kinesins [122].

### FRET

**Figure 3:**
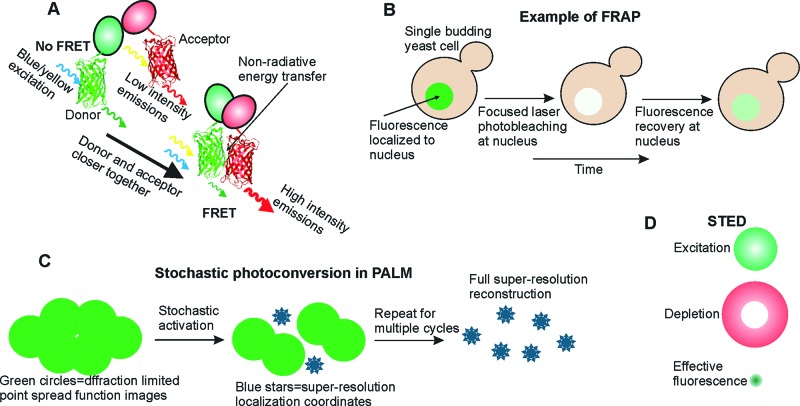
Examples of methods used in single-molecule studies (**A**) **FRET principle** based on the non-radiative energy transfer which occurs when donor and acceptor dye pairs (often, but not exclusively, fluorophores) are positioned within typically less than approximately 10 nm of each other (explanation is provided in the text); (**B**) **FRAP** illustrating photobleaching of fluorophores in a delimited region of a biological sample, here shown with a single budding yeast cell in which fluorescently labelled material in the nucleus is photobleached, followed by the measurement of fluorescence recovery over time; (**C**) **SMLM techniques (here exemplified with PALM)** illustrating selective activation of fluorophores and the final image after multiple photoactivation cycle repeats; and (**D**) **STED** showing excitation and depletion laser beams, and the effective fluorescence profile following stimulated depletion. Abbreviations: PALM, photoactivated localization microscopy; SMLM, single molecule localization microscopy; STED, stimulated emission depletion.

FRET is one of the most commonly used techniques to study putative interactions between neighbouring molecules. FRET utilizes the principle of non-radiative energy transfer between a donor and an acceptor molecule, which are often, but not exclusively, fluorescent (Figure 3A). If these molecules are close enough, typically separated by less than approximately 10 nm, then a donor being in an excited electronic state can transfer its excitation energy to an acceptor through electronic resonance of molecular orbitals [123,124]. This technique is commonly used in the study of a range of molecular interactions, in particular protein–protein and protein–nucleic acid. Temperature-dependent conformational changes of proteins, protein folding on a millisecond range, as well as dynamics of intrinsically disordered proteins, are possible to determine by combining FRET with a confocal microscopy excitation mode [125]. For example, confocal single-molecule FRET was used for determination of conformational changes in the *Listeria monocytogenes* P-type ATPase, LMC1, during its functional cycle [126].

Application of single-molecule FRET has been used in a range of protein-based molecular motors which act on DNA. One recent example of this is the replicative DNA helicase. Bell et al. from Howard Hughes Medical Institute, monitored opening and closing of *S. cerevisiae* ring-shaped Mcm2–7 DNA helicases during recruitment of the prereplicative complex to the origin of replication. These observations provided novel insights into the mechanism of replication initiation and quality control [127]. FRET studies on chicken Werner syndrome ATP-dependent helicase (WRN helicase) revealed a mechanism of DNA unwinding by WRN. Thus, this helicase unwinds DNA in a repetitive manner: repetitive DNA unwinding by WRN which happens on forked, 3′/5′-overhanging, G4 containing DNA substrates, although results of this phenomenon do depend on a DNA substrate [128]. Single-molecule FRET has been actively used in studies of DNA origami to obtain information about molecular structure and dynamics [129]. An energy transfer on a DNA origami substrate was visualized by using four-coloured FRET [130].

FRET-based biosensors have been only very recently developed but have rapidly become a widely used tool in the study of protein dynamics. One of the applications is the use of FRET-based sensors for determination and quantification of biomolecular crowding in live cells as an indicator of the physicochemical state of the cytoplasm [131]. Mechanical tension FRET sensors can also be used to study extra- and intracellular single-molecule force measurements [132]. DNA FRET sensors combined with TIRF imaging have been used to monitor DNA synthesis in real time where a simple setup utilizes a DNA primer labelled with a fluorophore acceptor and annealed to the DNA template which is labelled with the donor fluorophore. Molecular conformational changes upon extension altered the FRET signal which acted as a metric for DNA synthesis [133].

Significant recent progress has been achieved in studies of amyloid assembly processes by using single-molecule FRET combined with pulsed interleaved excitation (PIE) and fluorescence lifetime imaging microscopy (FLIM). Through reconstruction of the donor, FRET and acceptor images, as well as obtaining various parameters like FRET efficiency and fluorescence lifetimes, this technique enables the dynamics of protein aggregation to be followed. Thus, a two-step nucleation mechanism for amyloid fibrils formation through oligomerization was revealed [134].

### FRAP

FRAP involves in essence photobleaching a region of a cell or tissue in which a specific fluorescently labelled component is localized, and then quantifying the extent of any recovery of fluorescence intensity in that region subsequently (Figure 3B). Since unbleached components from outside this bleached area would have had to diffuse back into this area, such fluorescence intensity recovery is a metric of molecular mobility and turnover processes. The pioneering development of FRAP emerged from Watt Webb’s lab in the 1970s [135]. FRAP is now an established tool for enabling identification of molecular transport parameters such as diffusion and velocity coefficients, as well as kinetic parameters where molecular binding and unbinding events are involved [135]. For example, the localizations of nuclear envelope transmembrane (NET) proteins and their translocation rates on the inner and outer nuclear membranes were resolved by applying single-point illumination single-molecule FRAP [136]. FRAP analysis was used to determine diffusion coefficients of stromal interaction protein 1 (STIM1), a calcium sensor and a selective ion channel Orai1 at ER–plasma membrane junctions, revealing the dynamics of STIM1–Orai1 interactions [137].

Binding dynamics were also determined for protein–DNA and protein–protein interactions, for example glucocorticoid and mineralocorticoid receptors binding to DNA [138] and interaction dynamics between STAT2 and USP18 participating in type I interferon signalling [139] respectively. FRAP is also one of the main techniques to study anisotropic molecular diffusion on a less than micrometre length scale. Similarly, FRAP and single-molecule tracking visualized by wide-field fluorescence microscopy can be used in diffusion measurements and microdomain structure identification of a cylinder-forming polystyrene-poly(ethylene oxide) diblock copolymer (PS-b-PEO) film [140].

### PALM/STORM

Photoactivated localization microscopy (PALM) and stochastic optical reconstruction microscopy (STORM) are far-field imaging approaches that detect fluorescence following a photoconversion process of the fluorophore, either photoactivation in the case of PALM or photoswitching in the case of STORM. PALM/STORM techniques are examples of single molecule localization microscopy (SMLM), though, as discussed in the section above on the key historical developments, localization microscopy can be applied to single fluorescent molecules without requiring a photoconversion process. Additionally, the critical difference between PALM and STORM is not the type of fluorophore as such but rather the sequenced compared with random activation. In most PALM applications, fluorophores are stochastically activated and then imaged, activated then imaged etc. in multiple cycles (Figure 3C). In most STORM applications, the activation and imaging happen simultaneously, which can significantly increase the rate of data collection. PALM type imaging is also extremely useful in single-particle tracking type approaches. One or a very small number of fluorophores are excited at a given time, thus, their diffraction-limited areas do not overlap (Figure 3C). Excitation cycles can be repeated until all locations of target molecules are detected, which then can be assembled into the final image [141–143]. Therefore, PALM and STORM provide a wide range of applications in various fields of studies. These types of SMLM techniques have been widely used in recent cancer research. Yiping Ciu’s group applied these methods in order to visualize and track exosomes in human breast and cervical cancer cells (SKBR3 and HeLa cell lines respectively). By applying indirect immunofluorescence labelling with organic dyes, e.g. Alexa Fluor 488 and Alexa Fluor 647, the group showed accumulation of exosomes in lysosomes as well as interactions of cancer-derived exosomes with normal cells [144].

Plasma membrane research also benefits from using SMLM methods. For example, STORM allows dynamic tracking of N- and O-linked glycans on the membrane of live mammary cancer cells [145]. PALM was applied in the studies of protein organization in the plasma membrane, in particular, organization of glycosylphosphatidylinositol-linked proteins [146]. This technique is also extensively used in studies of protein oligomerization. For example, PALM revealed a multimeric organization of the Raf serine/threonine kinase which regulates cell growth through MAPK cascades [147]. Single molecules of G-protein–coupled receptor which become organized into dimers and higher oligomers were also visualized by PALM [148]. PhotoGate is an alternative to the PALM technique, which uses the principle of photobleaching of fluorescent particles and controls the number of fluorophopres that enter the region of interest. Fluorescent particles that arrive at the region of interest are repeatedly photobleached, thus, the concentration of fluorophores in the region of interest remains constant. Hence, PhotoGate does not require the use of photoconvertible fluorescent proteins but allows longer time for tracking single particles tagged with traditional fluorescent proteins (eGFP, mNeonGreen). Thus, this method enabled observation of monomer–dimer transitions of EGF receptor on a cell membrane as well as its intracellular signalling mediator, APPL1, interactions with early endosomes [149].

Combinations of PALM with other techniques allow studies of protein–protein interaction on a nanoscale level. A valuable method for probing protein–protein interactions is biomolecular fluorescence complementation (BiFC). In BiFC, one of the interacting proteins is labelled with a truncated version of a fluorescent protein, which is not fluorescent. A putative interacting protein can be labelled with the remaining truncated part of the fluorescent protein which similarly on its own is not fluorescent. If the two proteins interact the two complementary parts of the fluorescent protein can bind together, thus, an intact fluorescent protein molecule is restored which may then be excited into fluorescence [150]. However, the intrinsic spatial resolution of the technique is still limited by the diffraction of light [151]. Therefore, simultaneous use of BiFC and a super-resolution technique like PALM can enable imaging with, at best, approximately nanometre spatial precision. For instance, BiFC-PALM in studies of Ras GTPase and interactions with its downstream effector Raf showed Ras/Raf complexes assembling on the cellular membrane [151]. A year later, the same group showed a novel mechanism involved in cell signalling where signal transduction through MAPK pathways activation is dependent on Ras–GTP dimerization [152].

PALM has been used in biomineralization studies to investigate roles of biosilica-associated proteins in biosilica morphogenesis. Photoconvertible fluorescent proteins, Dendra2, mEos3.2 and Dronpa, were fused to biosilica-associated protein Silaffin-3 of model diatom *Thalassiosira pseudonana* [153].

Further development of SMLM methods has transformed them into techniques capable of obtaining multidimensional data. 3D information provides important insight into the architecture of molecules and systems, shedding new light on their structure and organization. For the first time, 3D PALM was used to visualize plasma membranes and integrin receptors within the ER [154]. Multicolour 3D STORM revealed molecular architectures of synapses in the brain, variations in their morphology, distribution and composition of neurotransmitter receptor [155]. Multidimensional imaging was applied to the studies of the cytoskeleton. For example, 3D PALM observations of live *Caulobacter crescentus* bacteria revealed organizations of a tubulin-like cytoskeletal protein FtsZ, providing direct evidence of its arrangement into Z-ring upon cell division [156]. 3D STORM using inclined illumination astigmatism imaging was employed in attempt to better understand the organization of F-actin [157]. 3D PALM has also been used in chromatin studies, for example to follow the distribution of the H2B histone, one of the core histones that form the nucleosome [158], and for imaging a budding yeast specialized H3 histone, Cse4 [159].

### STED

STED microscopy method, as discussed earlier, was experimentally demonstrated first by Stefan Hell, reported in 1994. This far-field method breaks down the diffraction limit through minimizing the area of excitation at the focal waist by controlled selective de-excitation of a target fluorophore. The focal plane is scanned by two laser beams (Figure 3D). The first one excites the fluorophores, the second beam of a longer wavelength is specifically altered such that at the focal plane has a donut shape. Therefore, only a small area from the centre of the donut shape is left to be able to emit the light. Thus the STED technique enables imaging below the diffraction limit [61,160].

Dual-channel STED for the first time allowed identification and characterization of signalling pathways involving astrocyte αvβ3 integrin and neuronal Thy-1 receptor, a cell adhesion molecule which is constantly expressed in the central nervous system. The capacity of STED to resolve Thy-1 clusters, with an effective diameter of 40–50 nm, revealed the involvement of this protein in the neuronal actin skeleton alterations [161]. A potential new role of the neuronal apoptosis inhibitory protein (NAIP), one of the proteins investigated in neurodegenerative disorders, was suggested after STED imaging of NAIP cellular localization upon cytokinesis [162].

STED microscopy can be also applied in various studies of the nucleus, such as chromatin studies via visualizing chromatin structure and characterization of architectural rearrangement invoked by physiological stimuli [163], looking at the spatial distribution of dsDNA repair factors [164] and replication factories [165], nuclear pore complex investigations via determination of NUP62 and NUP214 distributions [166].

There are a number of variants of STED approaches which all comprise light-induced transitions between at least two molecular states (e.g. bright and dark), one of which is fluorescent. A biologically valuable example of one of these STED variants is called reversible saturable/switchable optical fluorescence transition (RESOLFT), which stands for reversible saturable/switchable optical (fluorescence) transitions [167]. Like STED, the RESOLFT resolution goes beyond the diffraction limit, hence, allows nanoscale precise microscopy studies. Recent applications of RESOLFT using reversibly switchable fluorescent proteins have enabled visualization of individual spines within living hippocampal brain tissue [168], vimentin filaments [169], keratin and a structural protein of the nuclear pore complex [170]. Another variant of STED, MINFLUX, which like PALM/STORM uses selective stochastic fluorophore switching on and off, has been developed recently. MINFLUX allows 1 nm precision of molecules located 6 nm apart. This technique was used to image DNA origami and demonstrate diffusion of 30S ribosome subunit in *E. coli* [171].

### 3D single-molecule fluorescence microscopy tools

Another super-resolution method includes structured illumination microscopy (SIM). SIM illuminates the sample with a spatially periodic, structured illumination pattern, typically a series of grid-like patterns illuminated in different acquisitions at different orientations relative to the sample. The image is then analysed in so-called Fourier or frequency space. Small features in ‘real’ space have high spatial frequency values in Fourier space. Normal optical microscopy is limited by the optical resolution at the highest spatial frequency value that can be resolved in Fourier space, however, the periodic features of the structured illumination pattern can interfere with the high spatial frequency components in the sample to produce a ‘beat’ signal in Fourier space, due to the so-called Moiré effect, whose absolute value is lower than the threshold upper limit set by the optical resolution limit. Therefore, this beat signal contains super-resolution information which goes beyond the standard optical diffraction limit [172]. SIM improves the resolution of conventional light microscopy by a factor of roughly two in all three (*x, y* and *z*) spatial dimensions, which makes it a powerful tool in various fields of studies, such as the morphology of erythrocytes [173], the 3D structures of liver fenestrations and sieve plates [174], and in discerning precise details of subdiffraction limit of cortical microtubules [175]. There are, however, still some issues with SIM in that the structured illumination pattern can also result in the appearance of artifacts on the image of the orenitation and spacing of the illumination grid used, depite image analysis algorithms which are applied to attempt to remove these subsequently.

Some of the recent 3D fluorescence imaging methods, including double-helix microscopy developed by the group of Moerner et al. [176], and astigmatism can be implemented as an addition to many currently developed fluorescence microscopes as the required equipment can often be placed between the objective lens and the camera with relatively minor expertise in optical alignment required [61]. For instance, single-molecule localization microscopy in combination with astigmatism imaging applied to formalin-fixed, paraffin-embedded (FFPE) breast cancer samples after immunostaining, has enabled the visualization of proteins in mitochondrial and nuclear membranes as well as a cellular membrane protein (oestrogen receptor Her2) which is overexpressed in one-fourth of the cells [177]. RNA polymerase movements along DNA during the transcription cycle were tracked using 3D super-resolution imaging [178]. 3D single-molecule localization microscopy and 3D STORM are used in imaging fixed brain tissues allowing identification of several proteins simultaneously [179]. Also, a novel technique entitled cryogenic optical localization (COLD) has been reported recently. COLD provides spatial information about molecules through resolving 3D positions of several fluorophores attached to a single protein [180]. Detailed description of 3D super-resolution single-molecule microscopy methods has been published recently [181], and we steer the reader to this review for more detailed information.

There have been other attempts to improve existing 2D techniques in order to obtain higher spatial dimension resolution. For example, lattice light-sheet (LLS) microscopy has been successfully used as a non-invasive 4D imaging technique (three spatial dimensions plus the dimension of time) of live cells which allows visualization of intracellular dynamics [182]. LLS microscopy has also been combined with point accumulation for imaging of nanoscale topography (PAINT) microscopy [183]. In PAINT, the sample is continuously targeted by fluorescent probes present in solution throughout the imaging process [184]. LLS-PAINT with various PAINT labels enables 3D multicolour imaging of DNA and intracellular membranes during cell division [183]. Other variants of PAINT, such as DNA-PAINT and Exchange-PAINT, enable 2D and 3D visualization of DNA nanostructures as well as kinetic studies of DNA binding using a single fluorescent dye [185,186].

## Future perspectives

Extensive use of single-molecule fluorescence microscopy techniques have enabled visualization of an enormous range of different biological processes which was previously restricted by traditional population ensemble average methods. Many of the biological processes studied are components of very basic and fundamental systems in the cell. However, they have been essentially invisible until now: long-standing ‘old’ problems that have been simply intractable until the arrival of modern single-molecule fluorescence microscopy methods. The new tools have provided novel insights into the basic mechanisms of live cells as well as the identification of previously unknown functions of molecules which are essential to life as we know it. Despite recent achievements in making the invisible visible, experimental limitations do remain. For example, the signal-to-noise ratio can still be improved, especially when very rapid imaging is required to address biological questions at the submillisecond time scale. There is still a need for novel fluorescent proteins with enhanced brightness and photostability which would increase the possible illumination times and thus enable longer observations of molecules and processes in which they partake. Similarly, fluorescent proteins significantly increase the overall size of the tagged protein construct, since a fluorescent protein is often of comparable molecular weight with the native protein itself, which might affect its natural molecular conformations and thus its physiological behaviour and function. Therefore, the next generation of fluorescent molecules we would hope might become much smaller in size or even disappear completely.

For example, attempts to use digital holography as a super-resolution microscopy technique have already been made [187,188], with label-free imaging rendering, for example promising structural details of the dynamic morphology of filaments which enable single swimming cells to be motile [189]. Another example of a label-free technique is an interferometric scattering microscopy (iSCAT) with a single-molecule precision, applied recently to a range of biological questions by the group of Kukura et al. [190,191]. iSCAT has now been used in studies of motor proteins dynamics [192] which enabled visualization of microtubule disassembly [193], uncovered unknown details of myosin-5 stepping mechanism [194], and provided novel insights into kinesin-1 stepping cycle [195].

At present, there is no unique, single technique which can enable the simultaneous visualization of proteins and their post-translational modifications, for example as occurs during signal transduction. It would, in principle, also be valuable if we were able to obtain data concerning the molecular conformational states of intrinsically disordered regions during protein–protein or protein–nucleic acid interactions. However, attempts to study molecular conformational changes upon mechanical stretching perturbations have already been made by combining single-molecule fluorescence microscopy techniques with non-fluorescence approaches. For example, Fernandez et al. have utilized TIRF and AFM simultaneously to study the dynamics of stretching and unfolding of ubiquitin protein domains [196]. Combinations of AFM and FRET were also applied in studies of HPPK (6-hydroxymethyl-7,8-dihydropterin pyrophosphokinase) conformation [197]. TIRF and AFM-based single-cell force spectroscopy were also used in non-mechanistic studies and revealed protein cluster formation of integrins and their recruitment of adhesome protein [198].

There is no doubt that scientific excellence in the development of novel biophysical tools and techniques will continue to push back the borders of our understanding of life’s complex processes much further than at present. Interdisciplinary science approaches, when appropriately funded, are the best way forward to achieve these new developments.

## Abbreviations

BiFC: biomolecular fluorescence complementation
CLSM: confocal laser scanning microscopy
COLD: cryogenic optical localization
EGF: epidermal growth factor
ER: endoplasmic reticulum
HILO: highly inclined and laminated optical sheet
iRFP: IR fluorescent protein
iSCAT: interferometric scattering microscopy
LLS: lattice light-sheet
PAINT: point accumulation for imaging of nanoscale topography
PALM: photoactivated localization microscopy
RESOLFT: reversible saturable/switchable optical fluorescence transition
SERT: serotonin transporter
SIM: structured illumination microscopy
SMLM: single molecule localization microscopy
STED: stimulated emission depletion
STIM1: stromal interaction protein 1
STORM: stochastic optical reconstruction microscopy
TIRF: total internal reflection fluorescence
TOCCSL: thinning out clusters while conserving stoichiometry
WRN helicase: Werner syndrome ATP-dependent helicase
